# A specific objective supplemental factor in evaluating acute mountain sickness: ΔHR in combination with SaO_2_

**DOI:** 10.1186/s40779-015-0055-0

**Published:** 2015-10-26

**Authors:** Ming Li, Ji-Hang Zhang, Guo-Xi Zhao, Shi-Zhu Bian, Xu-Bin Gao, Xi Liu, Jie Yu, Jun-Qing Dong, Guo-Zhu Chen, Hong Wang, Lan Huang

**Affiliations:** Institute of Cardiovascular Diseases of PLA, Xinqiao Hospital, Third Military Medical University, Chongqing, 400037 China; Department of cardiology, 533 Hospital of PLA, Kunming, 650000 China; Department of Cadre Wards, Kunming General Hospital of Chengdu Command, Kunming, 650032 China

**Keywords:** Acute mountain sickness, Oxygen saturation, Heart rate difference

## Abstract

**Background:**

So far, there have been no measurements confirmed useful in diagnosing acute mountain sickness (AMS). The aim of this study was to determine the role of heart rate (HR) difference (**Δ**HR) and oxygen saturation ( SaO_2_) as objective risk factors in aiding the diagnosis of AMS.

**Methods:**

A total of 1,019 participants were assigned to either the acute exposure group (AEG): from 500 m to 3,700 m by flight within 2.5 h (*n* = 752); or the pre-acclimatization group (PAG): ascended to 4,400 m from 3,650 m within three hours by car after adapting 33 days at 3,650 m (*n* = 267). The questionnaires or measurements of resting SaO_2_ (oxygen saturation) and HR were completed between 18 and 24 h before departure and after arrival.

**Results:**

Incidence of AMS was 61.3 % (461) in AEG, with 46.1 % (347) mild cases and 15.2 % (114) severe cases. In PAG, the incidence was 38.9 % (104), with 30.7 % (82) mild cases and 8.2 % (22) severe cases. The AMS subjects showed a significant increase in HR and a decrease in SaO_2_ levels compared with the non-AMS subjects in both groups. **Δ**HR and post-exposure SaO_2_ were significantly correlated with the Lake Louise Score (LLS) in both groups. Stepwise logistic regression analysis revealed the **Δ**HR >25 and SaO_2_ < 88 % in AEG as well as **Δ**HR >15 and SaO_2_ < 86 % in PAG to be independent risk factors of AMS. Combining these two measurements could specifically indicate participants with AMS, which showed a positive predictive value of 89 % and specificity of 97 % in AEG as well as 85 % and 98 % in PAG.

**Conclusion:**

**Δ**HR or SaO_2_, as objective measurements, correlate with AMS. Combination of these two measurements may be useful as an additional specific and objective factor to further confirm the diagnosis of AMS.

## Background

Travelers may experience acute mountain sickness (AMS) due to the hypobaric hypoxia that occurs when individuals are exposed acutely to high altitude (above 2,500 m) or after pre-acclimatization to the same [1, 2]. AMS is a syndrome of non-specific symptoms including headache, gastrointestinal upset, fatigue, dizziness or insomnia that has become an important public health issue for highland newcomers [3, 4]. If these symptoms are ignored or the adaptation process fails, AMS may progress to more severe fatal diseases such as high altitude pulmonary edema (HAPE) or high altitude cerebral edema (HACE). In addition, it has been suggested that AMS represents a precursor of HAPE or HACE [2, 5]. Currently, AMS is mainly diagnosed by the Lake Louise Score (LLS), a subjective symptom questionnaire, which was established at the International Hypoxia Symposium at Lake Louise, Canada, 1993 [6]. This diagnosis is not objective and could increase the probability of misdiagnosis. As a result, for undiagnosed AMS, proper treatment including medicines, oxygen and optimized work plans cannot be used in time to avoid the risk of AMS progression and to maintain work efficacy. For other conditions with similar presentations, this may lead to a delay in treatment or even unnecessary death. However, the diagnosis of AMS is clinical and different measurements can only support it, yet there have been no measurements confirmed useful in diagnosing AMS so far. Hence, finding an objective aided evaluation system for AMS is crucial.

Many attempts have been made to find physiological parameters for evaluating AMS. Given that pulse oximetry is a commonly used, noninvasive means of assessing arterial blood oxygenation, some studies have focused on this means, in hopes of supporting the assessment of AMS and have shown that the presence of AMS is significantly associated with depressed oxygen saturation (SaO_2_) or elevated resting heart rate (HR) [5, 7–9]. Our previously published data have also shown HR and SaO_2_ as critical compensatory regulation factors of systemic oxygen delivery (DO_2_), correlated with AMS [10]. However, these measurements have not been confirmed useful in supporting the diagnosis of AMS. First of all, the exact nature of the correlation between HR, SaO_2_ and AMS has yet to be fully elucidated, and the cutoff for quantifying AMS at a given altitude is not available. Secondly, resting HR varies widely. In contrast, the difference  of HR between pre-exposure and post-exposure (ΔHR, post minus pre-exposure) may be more closely related to the presence of AMS [8]. In addition, most of the earlier studies were focused on the effect of a single parameter, but the dynamic and complex nature of AMS limits its utility in the evaluation of disease. Therefore, combining some relevant measurements seems to be more valuable. A predictive index has been proposed by combining clinical and hematological parameters of impending AMS [11]. However, the detection of hematological parameters is invasive. Clearly, further research is needed to establish an objective and simple noninvasive method to aid the evaluation of AMS.

In this study, we sought to clarify the association between AMS and ΔHR, SaO_2_ as well as to determine their roles, respectively, or in combination in aiding the evaluation of AMS in the cases of acute and pre-acclimatized exposure to high altitude.

## Methods

### Participants

The Ethical Review Board of the Third Military Medical University approved this study. Study participants were recruited from Chinese young men living at 500 m. Subjects with known cardiovascular or lung disease, active infection, or history of exposure to altitude above 3,000 m in the previous three months were excluded from the study. As a result, a total of 1,019 participants with an average age of 23 ± 4 years and a mean body mass index of 21.6 ± 2.1 kg/m^2^ were enrolled. All participants signed the informed consent. Subjects were assigned to two different groups: acute exposure group (AEG), 752 participants, traveled from 500 m to 3,700 m by flight within 2.5 h; another group, the pre-acclimatization group (PAG), composed of 267 participants, adapted 33 days at an intermediate high altitude of 3,650 m and then ascended to the destination altitude of 4,400 m within three hours by car.

### Questionnaire and measurement

Structured questionnaires were constructed with the Lake Louise Questionnaire scoring system and demographic information (age, weight and height). The questionnaires were completed under the guidance of experienced physicians, between 18 and 24 h after arrival at the destination. Resting SaO_2_ and HR were measured by Finger Pulse Oximetry (Nonin Onyx^®^ 9500; Nonin Medical, Inc.; Plymouth, MN, USA) between 18 and 24 h before departure and after arrival. Participants did not engage in any physical activity between arrival at high altitude and study completion. Before the study, participants were isolated from auditory and visual stimuli. Two parameters were measured in triplicate after the subjects had rested in a seated position for 15 min. More than 30 colleagues performed the measurement to ensure it could be done on time. In this study, AMS was diagnosed as the presence of headache with LLS ≥3, and the severity of AMS was defined as follows: three to five indicated mild AMS and six or more points indicated severe AMS [5, 12].

### Statistical analysis

Statistical analyses were conducted using SPSS V16.0 for windows software. To evaluate the differences between AMS and non-AMS groups, the Mann–Whitney U-test was applied to compare mean HR and SaO_2_ (non-normally distributed variables). The chi-squared test was performed for analysis of AMS incidence (enumeration data). To determine the risk factors for AMS, according to the method mentioned by Martin Burtscher’s study [13], we used the mean of potentially relevant risk factors with or without AMS as cutoffs, transformed them into dummy variables, and then analyzed them by backward stepwise logistic regression using Wald statistic. The criterion for statistical significance was *P* < 0.05. Data were presented as the mean ± SD.

## Results

### Distribution of demographic date, incidence and severity of AMS

The characteristics of the demographic data are presented in Table 1. None of the participants had a clear history of AMS, known cardiovascular or lung diseases, or used a preventive medicine such as acetazolamide. The distributions of age, body mass index (BMI), and ethnicity of participants with or without AMS were not different. The percentage of smokers was marginally lower in AMS compared with non-AMS, but the difference was not significant. Interestingly, when combining two groups together, the Chi-squared test showed smoking was a significant advantage at high altitude and correlated significantly with non-AMS (*P* = 0.004).

**Table 1:** Distribution of demographic data and clinical parameters between AMS and non-AMS

Parameter	AEG (*n* = 752)			PAG (*n* = 267)		
	AMS (*n* = 461)	Non-AMS (*n* = 291)	*P*-value	AMS (*n* = 104)	Non-AMS (*n* = 163)	*P*-value
Age (year)	23.0 ± 3.9	22.8 ± 3.9	0.475	22.2 ± 2.5	21.8 ± 2.3	0.162
BMI (kg/m^2^)	21.7 ± 2.2	21.8 ± 2.0	0.496	21.2 ± 1.8	21.4 ± 2.6	0.303
Han/ethnicity (%)	87.0 %	88.3 %	0.856	87.7 %	88.9 %	0.737
Medicine use (%)	0 %	0 %		0 %	0 %	
Smokers (%)	36.9 %	44.7 %	0.058	36.5 %	47.9 %	0.067
LLS	4.8 ± 1.7	1.6 ± 1.1	0.000	4.5 ± 1.6	1.2 ± 1.2	0.000
HR (pre-exposure)	65.7 ± 9.6	65.7 ± 9.6	0.978	75.6 ± 9.8	78.3 ± 10.6	0.091
HR (post-exposure)	86.5 ± 12.1^a^	82.2 ± 10.9^a^	0.003	86.8 ± 12.5^a^	85.1 ± 10.9^a^	0.487
**△**HR (post-pre)	20.9 ± 11.7	16.6 ± 10.6	0.009	11.1 ± 12.0	6.9 ± 9.0	0.010
SaO_2_ (pre-exposure)	98.10 ± 1.00	98.12 ± 1.02	0.436	91.60 ± 2.31	91.82 ± 2.30	0.45
SaO_2_ (post-exposure)	88.35 ± 3.23^a^	89.38 ± 2.76^a^	0.008	86.52 ± 2.60^a^	87.67 ± 2.82^a^	0.007
**△**SaO_2_ (pre-post)	9.75 ± 3.31	8.73 ± 2.89	0.008	5.08 ± 3.13	4.15 ± 2.86	0.010

*AMS* Acute mountain sickness, *AEG* Acute exposure group, *PAG* Pre-acclimatization group, *LLS* Lake Louise Score, **△** *HR* The difference of HR between pre-exposure and post-exposure, **△** *SaO*_2_ The difference of SaO_2_ between pre-exposure and post-exposure, *Pre-post* Pre-exposure minus post-exposure, *Post-pre* Post-exposure minus pre-exposure. ^a^ *P* < 0.01 compared with pre-exposure

**Fig. 1:**
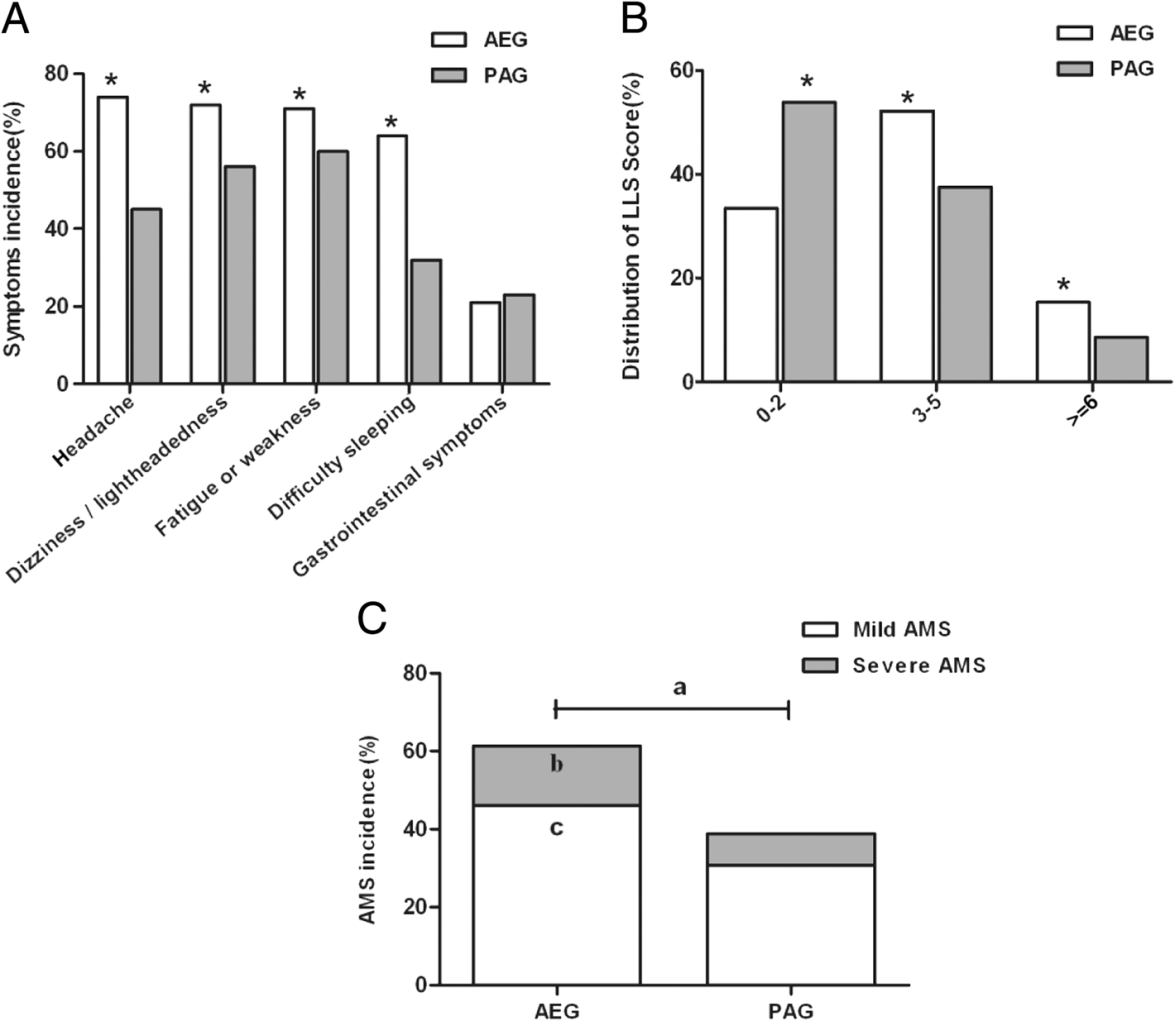
The distribution of symptoms or LLS and the incidence of AMS in AEG and PAG. AMS, Acute mountain sickness; AEG, Acute exposure group; PAG, Pre-acclimatization group. The comparison was made between AEG and PAG; *: *P* < 0.01; **a** *P* < 0.01 compared with total AMS; **b** *P* < 0.01  compared with severe AMS; **c** *P*  < 0.01  compared with mild AMS

The distribution of symptoms or LLS and the incidence or severity of AMS in all subjects are shown in Fig. 1. The incidence of AMS symptoms except gastrointestinal symptoms were significantly higher in AEG compared with PAG: headache (74 % vs 45 %), dizziness (72 % vs 56 %), fatigue (71 % vs 60 %) and difficulty sleeping (64 % vs 32 %). LLS in AEG was mainly distributed in the intermediate point section (51.2 % with 3–5 points), while, in PAG, it was mostly distributed in the low point section (53.9 % with 0–2 points). With regard to the incidence or severity of AMS, 61.3 % (461 of 752) participants in the AEG were diagnosed with AMS by the Lake Louise Score System, of which, 46.1 % had mild AMS and 15.2 % was severe. Whereas, in PAG, the incidences of mild, severe and total AMS were significantly lower: only 38.9 % (104) had AMS (mild 30.7 %, 82; severe 8.2 %, 22). Of note, none of the participants in either group was diagnosed with high altitude pulmonary edema (HAPE) or high cerebral edema (HACE).

### HR and SaO_2_ responses

The comparisons of HR and SaO_2_ in participants with or without AMS in AEG or PAG are presented in Table 1. The pre-exposure HR and SaO_2_ did not differ significantly between AMS and non-AMS participants. ΔHR was higher in subjects with AMS than without AMS in both groups. Post-exposure mean HR of the AMS subjects (86.5 ± 12.1 beats/min) was significantly higher than that of non-AMS subjects (82.2 ± 10.9 beats/min) in AEG but not in PAG. In addition, the AMS subjects in both groups also showed a significant decrease of SaO_2_ from pre-exposure (ΔSaO_2_, pre-post) and lower post-exposure mean SaO_2_ levels compared with subjects without AMS. The correlation between various physiological parameters and LLS in all subjects is shown in Table 2. ΔHR, ΔSaO_2_ and post-exposure SaO_2_ were significantly correlated with LLS among the study participants in both groups, while the post-exposure mean HR was associated with LLS only in AEG but not in PAG.

**Table 2:** Correlation between LLS and various physiological parameters in all subjects

Parameter	AEG		PAG	
	Spearman’s Rho	*P*-value	Spearman’s Rho	*P*-value
HR (pre-exposure)	0.001	0.973	−0.075	0.222
HR (post-exposure)	0.177	0.000	0.035	0.572
**△**HR (post-pre)	0.186	0.000	0.128	0.021
SaO_2_ (pre-exposure)	−0.043	0.239	−0.008	0.895
SaO_2_ (post-exposure)	−0.171	0.000	−0.183	0.003
**△**SaO_2_ (pre-post)	0.156	0.000	0.147	0.016

*AEG* Acute exposure group, *PAG* Pre-acclimatization group, **△** *HR* The difference of HR between pre-exposure and post-exposure, **△** *SaO*_2_ The difference of SaO_2_ between pre-exposure and post-exposure, *Pre-post* Pre-exposure minus post-exposure, *Post-pre* Post-exposure minus pre-exposure

Variables with a *P*-value of 0.10 or less were considered as potentially relevant risk factors for AMS. We used the mean of HR and SaO_2_ with or without AMS as cutoffs and transformed them into dummy variables, then analyzed them by backward stepwise logistic regression. The results including selected variables in the model and the odds ratio (*OR*) were shown in Table 3. From the results of the regression analysis, in AEG, ΔHR > 25 and SaO_2_ < 88 % were revealed to be independent predictors of AMS. Likewise, in PAG, a person with ΔHR > 15 showed two-fold more risk of suffering AMS than persons with small ΔHR (*OR* = 2.39, 95 % CI 1.34-4.26, *P* < 0.01). SaO_2_ < 86 % also increased the *OR* for AMS (*OR* = 2.86, 95 % CI 1.89-5.89, *P* < 0.01).

**Table 3:** Selected variables and *OR* of acute mountain sickness determined by stepwise logistic regression analysis

Selected variables	*P*-value	*OR*	(95 % CI)
AEG			
**△**HR (>25 vs ≤17)	<0.01	1.86	(1.24-2.79)
SaO_2_ (<88 % vs >90 %)	<0.01	1.72	(1.24-2.38)
HR (>87 vs >82)	0.08	1.38	(0.97-1.97)
PAG			
**△**HR (>15 vs ≤7)	<0.01	2.39	(1.34-4.26)
SaO_2_ (<86 % vs 86 %-88 %)	0.06	1.86	(0.98-3.57)
SaO_2_ (<86 % vs >88 %)	0.03	2.86	(1.39-5.89)

*AEG* Acute exposure group, *PAG* Pre-acclimatization group, **△** *HR* The difference of HR between pre-exposure and post-exposure

### AMS assessment with △HR and SaO_2_

**Fig. 2:**
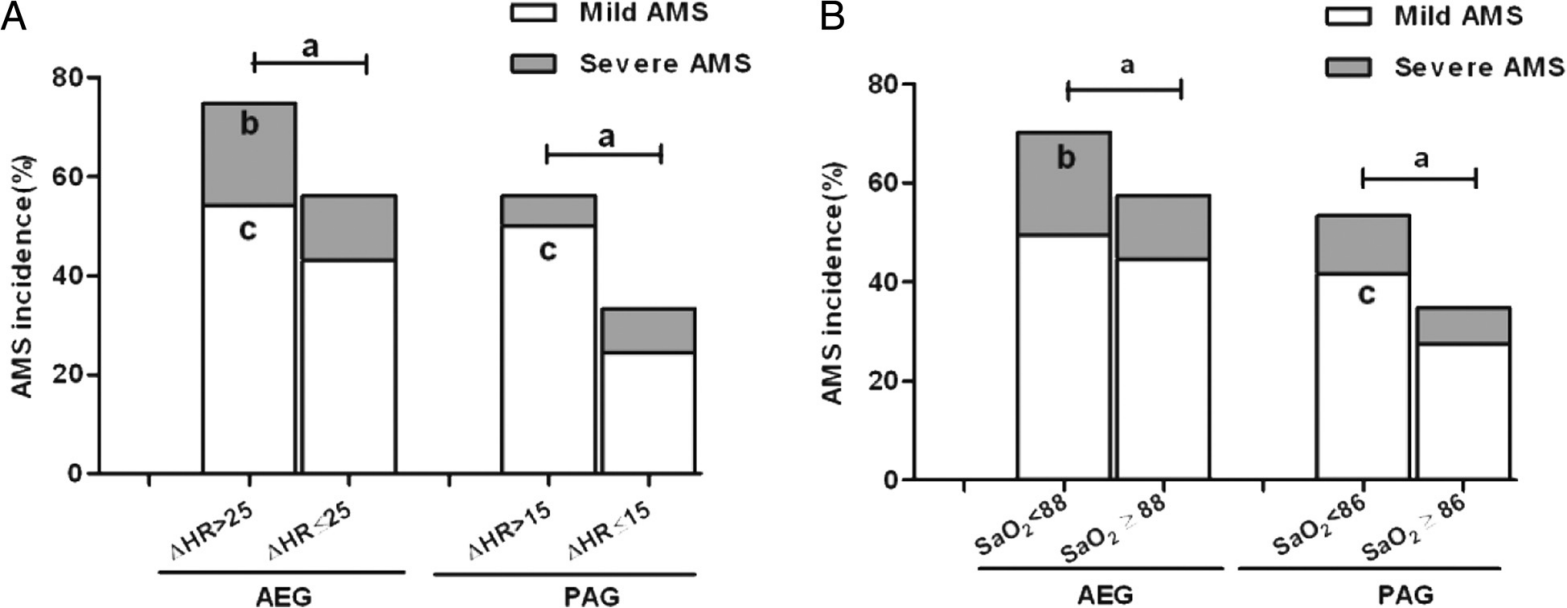
Effect of **△**HR cutoff (**a**) or SaO_2_ cutoff (**b**) on the AMS incidence. Comparison of AMS incidence between over and below cutoff values of **△**HR (**a**) or SaO_2_ (**b**) in AEG and PAG; *a*: *P* < 0.01 compared with total AMS; *b*: *P* < 0.01 compared  with severe AMS; *c*: *P* < 0.01  compared with mild AMS

Because ΔHR > 25 and SaO_2_ < 88 % in AEG as well as ΔHR >15 and SaO_2_ < 86 % in PAG were revealed to be independent risk factors of AMS, we used these cutoff values to evaluate the incidence and severity of AMS. As shown in Fig. 2, in the AEG, when ΔHR > 25 or SaO_2_ < 88 %, the incidence of severe and total AMS significantly increased (*P* < 0.01). In the PAG, a person with ΔHR >15 or SaO_2_ < 86 % also exhibited an obviously higher incidence of AMS except severe AMS. Furthermore, the ability to evaluate AMS with these cutoff values is demonstrated by calculating the sensitivity, specificity, positive and negative predictive values (Table 4). In the AEG, both ΔHR >25 and SaO_2_ < 88 % had a certain ability to evaluate AMS. In particular, the combination of these two factors increased specificity up to 97 % but decreased sensitivity to 16 %. These values cause a positive predictive value of 89 % and a positive likelihood ratio of 5.33 (Table 4). Similarly, in the PAG, combining ΔHR > 15 and SaO_2_ < 86 % gives a sensitivity of 16 % and a specificity of 98 %, which gave a positive predictive value of 85 % and a positive likelihood ratio of 8.0 (Table 4).

**Table 4:** The assessment test with the cutoff of △HR or SaO_2_ for AMS

Cutoff		AMS	Non-AMS	*N*	Sensitivity (%)	Specificity (%)	PPV (%)	NPV (%)
AEG								
SaO_2_ < 88 %	Yes	160	68	228	0.35	0.77	0.70	0.43
	No	301	223	524				
**△**HR > 25	Yes	155	52	207	0.34	0.82	0.75	0.44
	No	306	239	545				
SaO_2_ < 88 % + **△**HR >25	Yes	73	9	82	0.16	0.97	0.89	0.42
	No	388	282	670				
PAG								
SaO_2_ < 86 %	Yes	33	27	60	0.32	0.83	0.55	0.66
	No	71	136	207				
**△**HR > 15	Yes	37	29	66	0.36	0.82	0.56	0.67
	No	67	134	201				
SaO_2_ < 86 % + **△**HR > 15	Yes	17	3	20	0.16	0.98	0.85	0.65
	No	87	160	247				

*AEG* Acute exposure group, *PAG* Pre-acclimatization group, **△** *HR* The difference of HR between pre-exposure and post-exposure, *PPV* Positive predictive value, *NPV* Negative predictive value

## Discussion

The present data revealed that ΔHR and SaO_2_ are objectively measured correlates of AMS. Combining these two measurements may be useful as additional specific and objective factors to confirm the presence of AMS.

The result of ΔHR was supported by several previous studies that showed that the higher resting HR was associated with the presence of AMS [8, 9]. However, in this study, ΔHR was more closely related to the presence of AMS compared to resting HR. This may be because resting HR is widely variable due to its vulnerability to interference, such as barometric pressure or air temperature. In addition, a major limitation of measuring resting HR and SaO_2_ are potential behavioral influences (stimulation, excitement, etc.). The mechanism behind the ΔHR and AMS association could be related to a physiologic adaptation to reduce oxygen pressure. To maintain oxygen delivery to tissues, regulatory response in systemic level was performed, which was mainly embodied by an increase in cardiac output. This increase is provided primarily by an increase in HR due to a decrease in stroke volume (SV) [14, 15]. Another possible explanation for this phenomenon is that an increase in sympathetic tone is partly relevant to AMS [16, 17]. As stated previously, the cutoff of ΔHR for evaluating AMS in AEG is 25, whereas in PAG it is 15. The "cutoffs" would likely be different for those not acclimatized vs. acclimatized at a given altitude and different at different altitudes. Thus, more work remains to be done to clarify these relationships.

We found that SaO_2_ correlates with the presence of AMS and could also provide some ability to evaluate AMS in both groups. It is consistent with many earlier studies of SaO_2_ and AMS [7, 8, 17,18]. Only a few investigations reported a cutoff of SaO_2_ for evaluating AMS. In Michael S. Koehel’s study, SaO_2_ of 86 % or greater has the potential to rule out AMS, which was given a negative predictive value of 92 % at 4,380 m [7]. Martin Burtacher’s study determined the altitude-dependent SaO_2_ regression equation for AMS [18]. However, no one has found a reliable cutoff of SaO_2_ as a positive indicator of AMS. In this study, we applied the mean of SaO_2_ in subjects with AMS as the cutoff, which only gave a moderate positive predictive value. This unimpressive result may be due to the fact that the SaO_2_ difference between AMS and non-AMS is small. Taken together, applying the mean as cutoff is conservative. A more sensitive cutoff remains to be found as well as the relationship between the cutoff and destination altitude in further studies.

As mentioned above, the assessment model made by combining ΔHR and SaO_2_ gives a positive predictive value of 85 % and a specificity of 97 % in AEG and a positive predictive value of 85 % and a specificity of 98 % in PAG. Our results indicated that it could be helpful in identifying AMS in the AEG or the PAG, even though the two groups experience different exposure styles and have a different drops in PIO_2_. Unfortunately, the ability to screen or rule out AMS was unimpressive due to its low sensitivity. This indicates that the complexity and dynamic nature of AMS cause the low sensitivity of this model. Another limitation of this approach is the need for an accurate baseline measurement of individual resting HR at their low altitude residence and a similar need for accurate, true resting HR and SaO_2_ in the high altitude environment. The inherent nature of a field study also affected this unimpressive finding. For example, measurement for a large sample could not be completed during a strictly narrow period of time. In addition, other unidentified factors, such as some blood markers, may be sensitive indicators of AMS. Nevertheless, although the sensitivity is very low, at high altitude the consequences of false positives are still minor; the measurement of ΔHR and SaO_2_ is simple and safe, and this model did specifically identify the AMS participants. Firstly, if a person scored with AMS by LLS, and ΔHR and SaO_2_ were above the cutoff value, it supports more specifically the AMS diagnosis. Secondly, if a person has high LLS with a bad headache and is vomiting, but his ΔHR is elevated less than the cutoff and/or his SaO_2_ is above the cutoff, it is also considered AMS, as the LLS is the primary criteria and high LLS does correlate with AMS. On the other hand, if a subject claimed not to be sick and his ΔHR and SaO_2_ exceeded the cutoffs, he should be asked more pointedly whether he has had any symptoms, and be suggested to decrease activity and/or to take some medicine. If possible, adding the ΔHR and SaO_2_ cutoff measures as additional categories to be scored along with the LLS categories (headache, dizzy, etc.) might prove to be better than the original LLS, but this is difficult to prove. Lastly, these study participants were recruited only from young men due to some difficulties. Women also widely take part in high altitude activities including their employment in most of the world's military activities. The absence of women is cited as a limitation in the usefulness of these findings which must be addressed in a further study.

## Conclusion

In conclusion, **Δ**HR or SaO_2_ do correlate with AMS and could be somewhat helpful to confirm the presence of AMS. Combining these two measurements could be proposed as a specific and objective supplemental factor in evaluating AMS.

## Abbreviations

AMS: Acute mountain sickness
AEG: Acute exposure group
LLS: Lack Louise Score
PAG: Pre-acclimatization group
HR: The difference of **△**HR between pre-exposure and post-exposure
**△**SaO_2_: The difference of SaO_2_ between pre-exposure and post-exposure
